# The role of RNA methylation in glioma progression: mechanisms, diagnostic implications, and therapeutic value

**DOI:** 10.3389/fimmu.2025.1583039

**Published:** 2025-05-21

**Authors:** Shao-Ze Zhang, Shao-Yan Liu, Meng-Die Cheng, Yin-Feng Zhang, Jia-Wei Tian

**Affiliations:** ^1^ Department of Emergency Internal Medicine, The Affiliated Hospital of Qingdao University, Qingdao University, Qingdao, Shandong, China; ^2^ Institute for Translational Medicine, The Affiliated Hospital of Qingdao University, College of Medicine, Qingdao University, Qingdao, China; ^3^ Department of Neurosurgery, Beijing Tiantan Hospital, Capital Medical University, Beijing, China; ^4^ Department of Hospital Infection of Management Department, The Affiliated Hospital of Qingdao University, Qingdao, Shandong, China

**Keywords:** epitranscriptome, M6A, m5C, m7G, m1A

## Abstract

Glioma represents a highly lethal form of malignant tumour, with RNA methylation emerging as a critical regulator of its oncogenesis and progression. As a prevalent post-translational modification, methylation influences various biological functions, particularly RNA processing, by modulating splicing, transport, and degradation of both mRNAs and noncoding RNAs. Key methylation types such as N6-methyladenosine (m6A), N5-methylcytosine (m5C), N7-methylguanosine (m7G), and N1-methyladenosine (m1A) are dynamically regulated by specific enzymes known as writers, erasers, and readers. Dysregulation of these modifications contributes to glioma pathophysiology, while offering potential biomarkers for early detection and promising therapeutic targets. This review explores the mechanistic roles of RNA methylation in glioma and highlights its translational implications, aiming to advance molecular diagnostics and targeted interventions in glioma treatment.

## Graphical Abstract

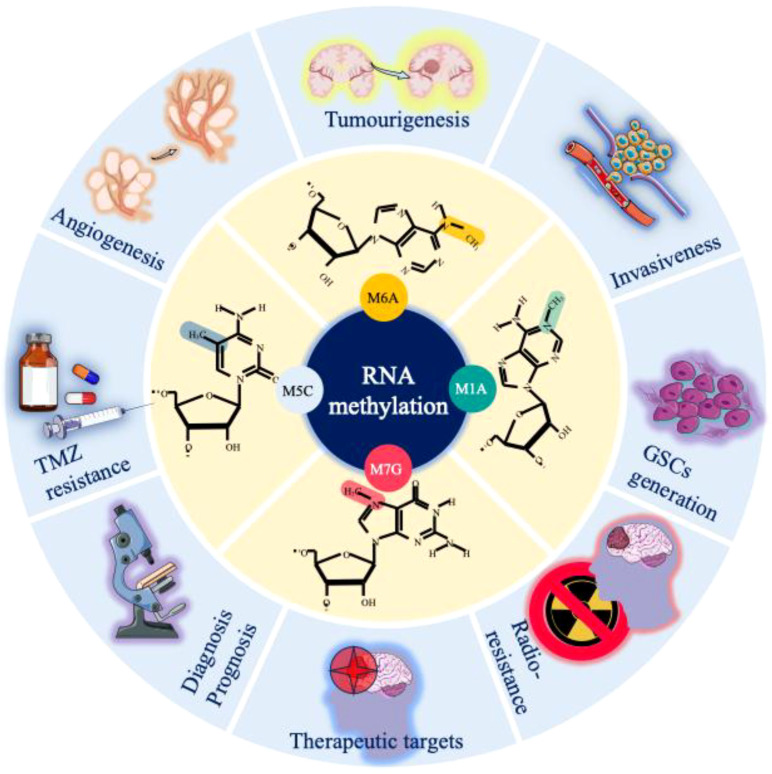

## 1 Introduction

**Figure 1:**
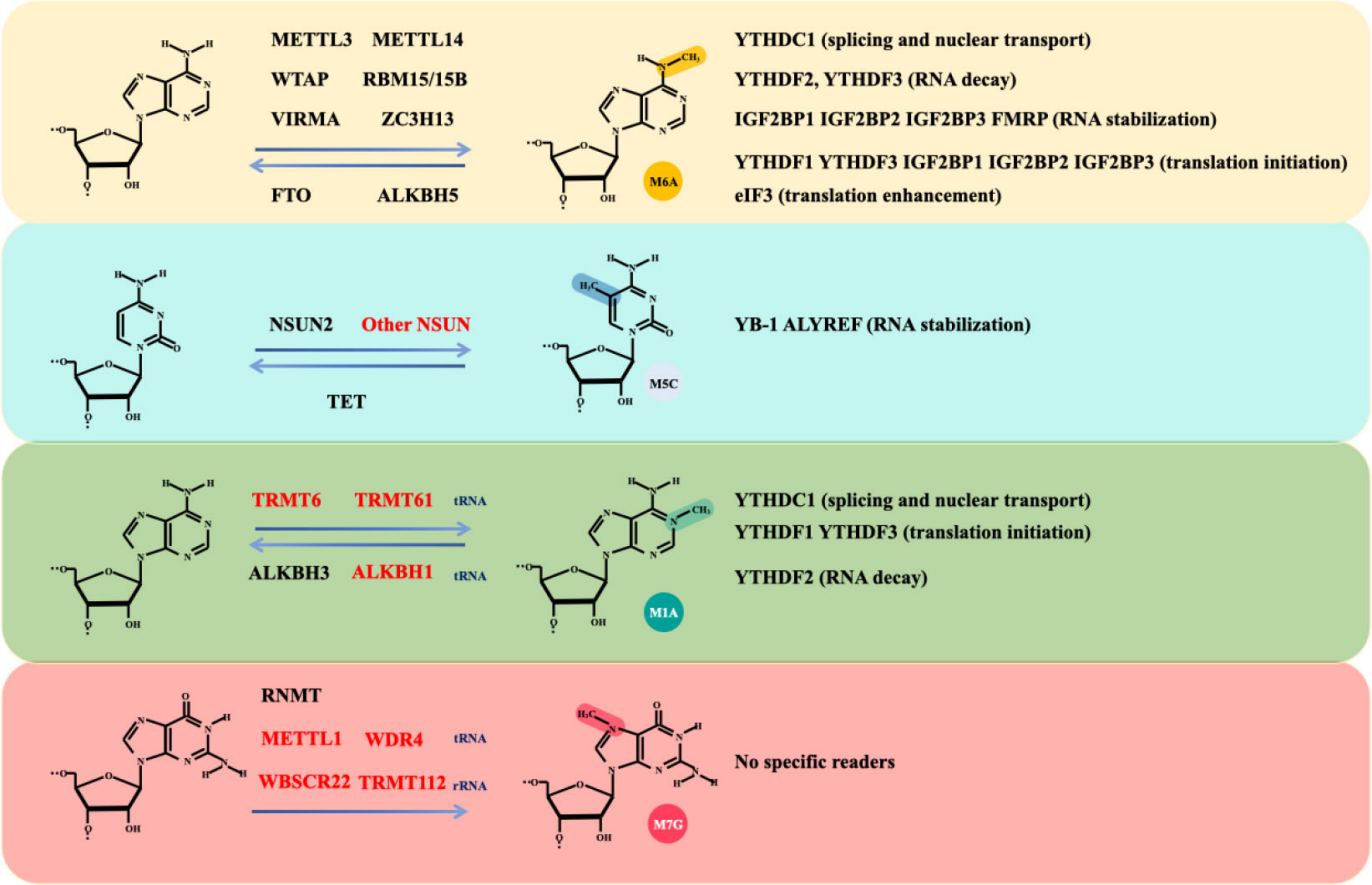
The main types of RNA methylation modifications. RNA methylation encompasses several modifications, including m6A, m5C, m7G, and m1A. The formulas demonstrate the dynamic and reversible processes of RNA methylation modifications and associated writers and erasers. Writers and erasers highlighted in red are specifically involved in noncoding RNA modifications, while others highlighted in black are involved in mRNA modifications. Different types of methylation modifications are recognized by distinct RNA readers, leading to various biological effects, including RNA decay, stabilization, splicing, nuclear transport, translation initiation and enhancement.

Glioma is the most common and lethal primary malignant tumour (14.2% of all tumours and 50.1% of all malignant tumours) of the central nervous system (CNS) (1). The annual incidence of glioma is approximately 6 per 100,000 individuals, with a male-to-female prevalence ratio of 1.6 (2). Gliomas are primarily categorized into four distinct groups: adult diffuse gliomas, paediatric diffuse low-grade gliomas, paediatric diffuse high-grade gliomas, and localized astrocytoma (3). Despite substantial investments in glioma-associated research, the underlying mechanisms governing glioma development remain inadequately understood. However, in recent years, with advancements in clinical and transcriptomic research, several crucial mechanisms underlying glioma progression have been elucidated, which have significantly contributed to the diagnosis and treatment of glioma. Among these modifications, RNA modification is a widespread and common posttranslational modification that is significantly altered during the development and progression of cancers, including glioma (4). Furthermore, RNA methylation is the most common type of RNA modification and widely occurs in various processes of the cell cycle. It plays an important role in the regulation of gene expression and RNA stability, thus affecting the occurrence and development of cancer cells (5). Methylation and its effects on RNA are collectively determined by methyltransferases (writers), demethylases (erasers), and RNA-binding proteins (readers). These methylation-associated proteins can significantly alter the splicing, export, translation, and decay of RNA (6). We briefly summarize the various types of methylation-associated proteins and their biological effects (**Figure 1**).

### 1.1 m6A

In the process of RNA methylation, N6-methyladenosine (m6A) modification has been the most frequently studied modification (7). Importantly, RNA modifications, particularly m6A modifications, have been shown to be essential for tumour development (8). The m6A modification of RNA is located primarily within the mRNA transcription start site and the 3′-untranslated region (UTR) and commonly occurs in the conserved RRACH (R, purine; H, nonguanine base; A, adenine; C, cytosine) motif (9, 10). The effects of m6A modification on mRNAs include splicing transcripts (11), epigenetic silencing (12), and stabilizing the mRNA, the latter of which is the main effect (13, 14). In addition, m6A modifications are present in other types of RNA such as ribosomal RNA (rRNA), transfer RNA (tRNA), small nucleolar RNA (snoRNA), microRNA (miRNA), long noncoding RNA (lncRNA), circular RNA (circRNA), and small nuclear RNA (snRNA) (15, 16).

The m6A writer refers to the methyltransferase complex (MTC). The MTC consists of two parts: the catalytic subunit m6A-METTL complex (MAC) and the regulatory subunit m6A-METTL-associated complex (MACOM). The methyltransferase-like 3 (METTL3) and methyltransferase-like 14 (METTL14) constitute the MAC, and the RNA-binding motif protein15/15B (RBM15/RBM15B), Wilms’ tumour 1-associating protein (WTAP), and VIR-like m6A methyltransferase associated (VIRMA) form the core structure of the MACOM (17, 18). ZC3H13, a zinc-finger protein, is integral to the regulation of RNA m6A methylation within the nucleus and is recognized as a crucial component of the m6A methylation complex (19). It promotes the nuclear localization of WTAP, Virilizer, and Hakai, thereby ensuring accurate m6A methylation of RNA by the MTC (19). Additionally, ZC3H13 interacts with VIRMA, inducing a conformational change in the latter (20).

The term m6A eraser refers to a demethylase. The fat mass and obesity-associated protein (FTO) and the α-ketoglutarate-dependent dioxygenase alkB homologue 5 (ALKBH5) demethylate RNA m6A residues through their oxidative demethylation activity (21, 22). FTO preferentially demethylates N6,2’-O-dimethyladenosine (m6A_m_) rather than m6A and reduces the stability of m6A_m_ mRNAs (23). m6A_m_ modification is a terminal cap modification found in higher eukaryotic mRNAs and ncRNAs, particularly snRNAs and viral RNAs, typically occurring at the first or occasionally second nucleotide following the m7G cap (24). This modification is consistent with the fact that FTO mainly catalyses snRNA methylation (23). ALKBH5, another demethylase, significantly affects mRNA export, RNA metabolism, and the assembly of mRNA processing factors within nuclear speckles (22).

Through the specific recognition and binding of m6A residues, m6A reader proteins are integral to a variety of biological processes, including RNA splicing, export, translation, and decay (6). Different families of readers recognize different m6A-modified RNAs, which mediate different physiological functions (7). Notably, the RNA methylation condition is determined by the relative weight of m6A writer or eraser functions, but most of the final biological effects of RNA methylation are determined by m6A readers. The m6A readers include the YTH domain family (YTHDF), YTH domain-containing family (YTHDC), insulin-like growth factor 2 mRNA-binding protein (IGF2BP), eukaryotic initiation factor 3 (EIF3), and heterogeneous nuclear ribonucleoprotein (hnRNP) (25). Readers are classified into nuclear readers and cytoplasmic readers based on their location. The nuclear readers include YTHDC1, YTHDF2, YTHDF3, Fragile X mental retardation protein (FMRP), and hnRNPA2/B1, which are responsible for the splicing and exporting of mRNAs. The cytoplasmic readers include YTHDF2, YTHDF3 (RNA decay), IGF2BP1, IGF2BP2, IGF2BP3, FMRP (RNA stabilization), YTHDF1, YTHDF3, IGF2BP1, IGF2BP2, IGF2BP3 (translation initiation), and EIF3 (translation enhancement) (25). Notably, YTHDC2 stands out as the largest m6A-binding protein within the YTH protein family and is distinguished by its exclusive possession of ATP-dependent RNA helicase activity (26).

### 1.2 m5C

N5-methylcytosine (m5C) modification is another RNA methylation process that specifically modifies the fifth carbon atom of cytosine. It is found mainly in cytoplasmic and mitochondrial mRNAs, enhancer RNAs, and several noncoding RNAs. Notably, the distribution of m5C on mRNAs is also not random. m5C modifications in mRNAs are enriched mainly in nontranscriptional regions (27).

Like m6A, m5C is regulated by writer, eraser, and reader proteins. The m5C writers include the DNA methyltransferase 2(DNMT2) and NOL1/NOP2/SUN domain (NSUN) families, which include 7 members (NSUN1-7) (28–31). Among these writers, DNMT2 are responsible for DNA m5C methylation via the catalysis of the covalent addition of methyl groups to the cytosine ring (29). Only NSUN2 is involved in mRNA modification and was identified by ALY/REF export factor (ALYREF) to mediate mRNA nucleus export (32, 33). The TET family is the main eraser of DNA m5C methylation and has three members: TET1, TET2, and TET3. TET1 and TET2 are distributed mainly in the nucleus, whereas TET3 is found in both the nucleus and the cytoplasm (34–36). The m5C readers include Y box binding proteins 1 (YB-1) (37) and ALYREF (38, 39). ALYREF is a subunit of the transcription-export complex (TREX), which mediates mRNA nuclear export and binds to its m5C sites in the 3’-UTR to prevent mRNA degradation (38, 39). Additionally, YB-1 stabilizes m5C-modified mRNAs and promotes the development of a variety of cancers such as digestive cancers, glioblastoma (37), and leukemia (40).

### 1.3 m7G

N7-methylguanosine (m7G) modification is another type of RNA methylation (41, 42). The m7G modification of RNA is a widespread posttranscriptional modification found in mRNAs, rRNAs, and tRNAs (43, 44). Researchers have noted that m7G modification of mRNAs is particularly abundant in the 5’UTR and in AG-rich contexts (44). Currently identified m7G writers include RNA guanine-7 methyltransferase (RNMT) and its cofactor RNMT-activated small protein (RAM), the methyltransferase 1-WD repeat-containing protein 4 (METTL1-WDR4) complex, the Williams-Beuren syndrome chromosome region 22 (WBSCR22) and tRNA methyltransferase activator subunit 112 (TRMT112) (45–48). Notably, these m7G writers exhibit different catalytic activities towards various RNA types. Specifically, RNMT/RAM is involved in mRNA methylation, contributing to the cap homeostasis of the mRNA transcriptome (45). The METTL1-WDR4 complex participates in the m7G modification of tRNA and prevents its degradation (46). WBSCR22 and TRMT112 are responsible for mediating m7G methylation in rRNA (47).

### 1.4 m1A

In addition, RNA modifications include N1-methyladenosine (m1A) modifications (49). In general, the functional role of m1A lies in promoting the translation of methylated mRNAs (50). m1A modification is dynamically regulated in mammalian RNAs. Among the m1A methyltransferases, the nucleomethylin (NML) and tRNA methyltransferase 10C (TRMT10C) methylate mitochondrial ND5 mRNA, whereas TRMT6 and TRMT61A methylate tRNA T-loop-like structures (51). TRMT61B methylates human mitochondrial tRNAs (52). In conclusion, NML, TRMT10C, TRMT6, TRMT61A, and TRMT61B are identified as m1A writers.

ALKBH1 and ALKBH3 are responsible for demethylation. ALKBH1 is a tRNA demethylase that mediates the demethylation of N1-methyladenosine (m1A) in tRNAs, which results in attenuated translation initiation and decreased usage of tRNAs in protein synthesis (53). Reversible tRNA modifications can broadly affect protein synthesis. However, the role of altered protein synthesis in the development of glioma remains to be further studied. ALKBH3 is the only known mRNA m1A demethylase. One study revealed that Thr133 is mutated to the corresponding residue, converting the selectivity of the ALKBH3 substrate from m1A to m6A (49). However, the role of ALKBH3 in the development of glioma has not yet been reported.

Similar to the m6A modification, YTHDF1, YTHDF2, YTHDF3, and YTHDC1 are considered methylation readers. However, the m6A reader YTHDC2 has no m1A binding activity (54). A previous study revealed that removing ALKBH3 increases the amount of m1A-modified mRNA. The endogenous transcripts modified by m1A can recognize the reader YTHDF2, which mediates its degradation (55). In addition, the comparison between LGG and HGG revealed that high expression of NML, TRMT6, TRMT10C, TRMT61B, ALKBH1, ALKBH3, YTHDF1, YTHDF2, and YTHDF3 are risk factors for HGG, whereas YTHDC1 is a protective factor (56).

In summary, RNA methylation involves a complex network in which writers, erasers, and readers interact with and influence each other, jointly regulating the cell developmental cycle and physiological functions (**Supplementary Table 1**). Disruption of any part of this network can trigger a series of downstream effects, potentially promoting the occurrence and progression of various tumours, including gliomas (37, 40). To gain a deeper understanding of the role of RNA methylation in gliomas, we have summarized the mechanisms of RNA methylation in the pathogenesis of gliomas. Furthermore, tracking and monitoring changes in methylation levels may aid in the early diagnosis of gliomas, and drugs developed to target this process have shown promise in the treatment of gliomas (4).

## 2 The milestones in RNA methylation field

**Figure 2:**
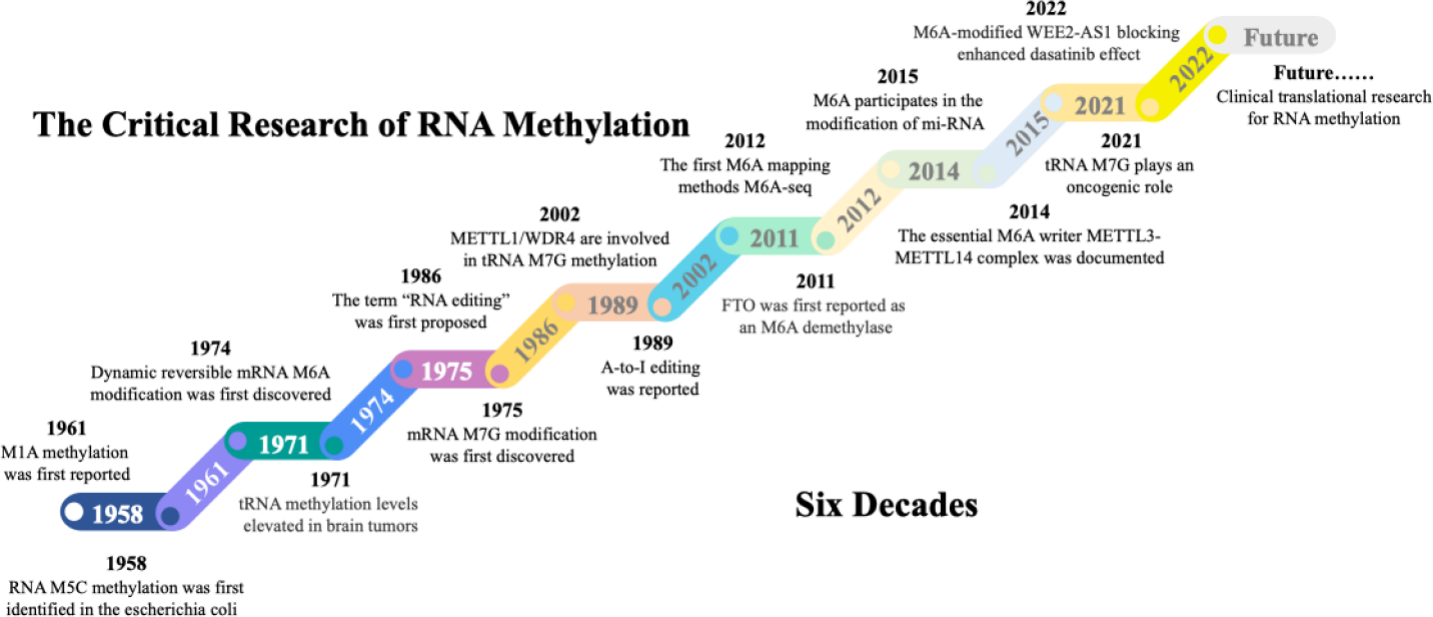
The milestones in RNA methylation field. The first RNA methylation type m5C was discovered from bacteria in 1958. Since then, other RNA methylation, including m1A, m6A, and m7G were progressively discovered. Along with the accumulation of knowledge on RNA modifications, regulators related to m6A, m5C, m7G, and m1A have been discovered, further completing the RNA editing and splicing theories. This lays the groundwork for further research into the mechanisms of methylation in disease development. The past six decades have witnessed significant advancements in mapping RNA methylation through epi-transcriptomic technologies and tremendous progress from the discovery of RNA methylation to its omics research. The future research direction for RNA methylation will focus more on its application in disease-oriented clinical studies to better improve patient outcomes.

The RNA synthesized following DNA transcription undergoes more than 170 posttranscriptional modifications (57). These modified RNAs include mRNAs, rRNAs, and tRNAs, and the modifications include mainly m6A, m5C, m7G, and m1A methylation. The discovery of these RNA methylation processes has undergone a considerable exploration (**Figure 2**). In 1958, m5C methylation was first discovered in RNA from *Escherichia coli (* 58). Shortly after that, m1A methylation was documented in the 1960s (59). In 1971, researchers reported that tRNA methylation levels are significantly elevated in brain tumour tissues (60). This paved the way for a new perspective and insight into the role of RNA methylation in the mechanisms underlying glioblastoma formation. In 1974, the dynamic reversible m6A modification of mRNAs was first discovered (61). Shortly thereafter, the initial function of m6A methylation was demonstrated, revealing a connection between the presence of m6A and mRNA instability (62). In 1975, the modifying effect of m7G on mRNA was reported (63). In 1986, the concept of “RNA editing”, which helps researchers better understand the molecular regulatory mechanisms within cells, was first proposed. Additionally, the discovery of the conversion of adenosine into inosine (A-to-I editing) in 1989 further confirmed the importance of posttranscriptional modifications. Since then, scientists have realized that in addition to DNA, posttranscriptional modifications play irreplaceable roles in determining biological traits and disease processes (64, 65). A 2002 report revealed that the m7G writers METTL1 and WDR4 were involved in tRNA m7G methylation; this was the first report of an m7G writer (48). In 2011, FTO was first reported as an m6A eraser that has demethylase activity (21). In 2012, the advent of antibody-mediated capture and massively parallel sequencing with the m6A-Seq technique led to the analysis of RNA methylation at the transcriptome-wide level (66). Moreover, based on the combination of m6A-specific methylated RNA immunoprecipitation and next-generation sequencing (MeRIP-Seq), researchers have reported that m6A sites are enriched near stop codons and in 3’-untranslated regions (3’UTRs) (67). In 2014, the METTL3-METTL14 complex was shown to function as an essential m6A writer, possessing specific methyltransferase activity for RNA m6A methylation (68). Since then, research on methylation writers has experienced a remarkable surge, particularly in the investigation of m6A methylation. A 2015 study revealed that m6A participates in the modification of primary miRNAs, which are recognized by the RNA-binding protein DGCR8 and trimmed into mature miRNA (69). Researchers have begun to realize that the role of noncoding RNA methylation in altering the biological functions of cells is as important as that of mRNAs and cannot be disregarded. In 2021, m7G modification was shown to participate in the modification of tRNAs and increase the translation of cell cycle regulator mRNAs, thus leading to oncogenic transformation. These findings underscore the comparable significance of noncoding RNA methylation and mRNA methylation in cancer development (70, 71). Those results also provide a new way to understand the occurrence and progression of cancers. With the deepening understanding of RNA methylation and the underlying mechanisms of cancer initiation, research on RNA methylation has increasingly transitioned from theoretical exploration to clinical applications. Notably, several RNA methylation-based antitumour drugs have exhibited promising therapeutic prospects. A 2022 study reported that the therapeutic sensitivity to dasatinib can be improved by blocking WEE2-AS1 expression (72). We anticipate a proliferation of diagnostic biomarkers and therapeutic drugs targeting RNA methylation alterations in the future. These advances offer great prospects for extending the human lifespan.

## 3 The roles of RNA methylation in glioma pathology

As research on RNA methylation has advanced, an increasing number of oncogenic roles related to RNA methylation have been elucidated (17). Notably, recent studies have revealed a close association between RNA methylation and the onset and progression of gliomas (14, 73, 74). Moreover, as investigations into glioma treatment have advanced, RNA methylation has been shown to play a role in the emergence of drug resistance in gliomas (75). Through a comprehensive review of the literature, we discovered that RNA methylation constitutes a highly intricate regulatory network (**Table 1**). Both excessive methylation and demethylation disrupt the delicate balance of methylation within cells, thereby triggering the onset and progression of gliomas. For a deeper understanding of the role of this RNA methylation regulatory network in gliomas, we summarize the mechanisms of RNA methylation in the genesis, development, and drug resistance of gliomas.

**Table 1:** Mechanisms of RNA methylation-associated proteins in glioma development and progression.

Methylation types	Rugulators	Expression alterations	Target RNAs	Readers/Pathways	Mechanisms	Phenotypes	Clinical implications	Refs
m6A	METTL3	Elevated	SOX2 mRNA	HuR	Enhancing SOX2 mRNA stability and promoting its expression	Promoting GSC maintenance, dedifferentiation, and aggressiveness	Tumour promoter	(13)
	METTL3	Elevated	SOX2 mRNA	HR pathway	Enhancing DNA-damage repair	Reducing sensitivity to gamma irradiation, promoting radio resistance	Tumour promoter	(13)
	METTL3	Elevated		RasV12	Enhancing astrocytes immortality	Tumour growth	Tumour promoter	(13)
	METTL3	Elevated	MALAT1 mRNA	HuR/NF-κB signalling	Stabilizing the MALAT1 mRNA and promoting its expression	Promoting the malignant progression of gliomas	Tumour promoter	(14)
	METTL3	Elevated	UBXN1 mRNA	YTHDF2/NF-κB signalling	Degrading the UBXN1 mRNA and reducing its expression	Promoting the malignant progression of gliomas	Tumour promoter	(74)
	METTL3	Elevated	SRSF mRNA	YTHDC1	Enhancing the SRSF mediated ASEs, inhibiting NMD	GBM tumour growth and progression	Tumour promoter	(83)
	METTL3	Elevated	BUD13 mRNA CDK12 mRNA		Stabilizing the BUD13 and CDK12 mRNA, enhancing their expression	Promoting the VM process of GBM, reducing sensitivity to anti-angiogenic drugs	Tumour promoter	(122)
	METTL3	Elevated	LINREP lncRNA	HuR	Stabilizing lncRNA LINREP, inhibiting PTBP1 degradation, impeding ASEs	Promoting the malignant progression of gliomas	Tumour promoter	(108)
	METTL3	Elevated	ADAR1 mRNA	YTHDF1	Stabilizing the ADAR1 mRNA, ADAR1 and promoting A-to-I editing	Tumour growth	Tumour promoter	(94)
	METTL3	Elevated	MGMT mRNA ANPG mRNA		Stabilizing the MGMT and ANPG mRNA and promoting their expression	Promoting the TMZ resistance of glioma	Tumour promoter	(134, 135)
	METTL3	Elevated	ADAM19 mRNA		Reducing the ADAM19 expression	Inhibiting GSCs growth and self-renewal	Tumour suppressor	(78)
	METTL3	Elevated	circDLC1 circRNA	circDLC1/miR-671-5p/CTNNBIP1 axis	Enhancing circDLC1 expression	Repressed the malignant proliferation	Tumour suppressor	(144)
	METTL3	Decreased	HSP90 mRNA		Enhancing the HSP90 expression	Inhibiting apoptosis, promoting glioma development	Tumour suppressor	(145)
	METTL14	Elevated	ADAM19 mRNA		Reducing the ADAM19 expression	Inhibiting GSCs growth and self-renewal	Tumour suppressor	(78)
	WTAP	Elevated	mRNA	CD97	Increasing the EGFR expression	Tumour invasiveness and migration	Tumour promoter	(117) (118)
	WTAP	Elevated	mRNA	PI3K-AKT and extracellular signal-related kinase pathways	Increasing the EGFR expression	Promoting cell proliferation, migration, and invasion but inhibit apoptosis in GSCs	Tumour promoter	(115)
	ZC3H13	Decreased	mRNA		Inhibiting localization of the ZC3H13-WTAP-Virilizer-Hakai complex in the nucleus	Increasing TMZ resistance	Tumour suppressor	(138)
	ZC3H13	Decreased	DUSP9 mRNA	ERK pathway	Reducing DUSP9 expression	Enhancing microglial M2 polarization, promoting GBM progression	Tumour suppressor	(98)
	FTO	Decreased	pri-miR-10a	hnRNPA2/B1	Inhibiting miR-10a, activating oncogenic pathway	Increasing the glioma tumour burden	Tumour suppressor	(142)
	FTO	Elevated	MYC mRNA	MYC signalling pathway	Enhancing the expression of miR155 and miR23a cluster, suppressing the MXI1 expression	Tumourgensis and drug resistance	Tumour promoter	(80)
	ALKBH5	Elevated	FOXM1 premRNA	HuR	Stabilizing the FOXM1 pre-mRNA and enhancing FOXM1 expression	Proliferation and tumourigenesis of GSCs	Tumour promoter	(79)
	ALKBH5	Elevated	ZDDHC3 mRNA	YTHDF2	Increasing PD-L1 protein expression	Suppressing TIME immune activation, promoting tumour growth	Tumour promoter	(96)
	ALKBH5	Elevated	G6PD mRNA	PPP	Stabilizing the G6PD mRNA, promoting G6PD translation and activating the pentose phosphate pathway (PPP)	Proliferation of glioma cells	Tumour promoter	(97)
	ALKBH5	Elevated	mRNA			EMT and VM	Tumour promoter	(123)
	ALKBH5	Elevated	NANOG mRNA		Stabilizing the NANOG mRNA and enhancing NANOG expression	Increasing TMZ resistance	Tumour promoter	(135)
	ALKBH5	Elevated	SOX2 mRNA	Wnt5α/β-catenin signalling pathway	Stabilizing the SOX2 mRNA and enhancing SOX2 expression	Promoting glioma cell proliferation, increasing TMZ resistance	Tumour promoter	(137)
	ALKBH5	Elevated	mRNA	HR pathway	Enhancing DNA-damage repair	Promoting radioresistance and invasiveness	Tumour promoter	(95)
	YTHDF2	Elevated	MYC mRNA VEGFA mRNA	YTHDF2-MYC axis	Stabilizing MYC and VEGFA transcripts, enhancing IGFBP3 expression	GSC growth	Tumour promoter	(82)
m5C	NSUN2	Elevated	ATX mRNA		Increasing the expression of ATX protein, converting the LPC into LPA	Promoting the migration and proliferation of glioma cells	Tumour promoter	(33)
	NSUN5		β-catenin mRNA	CD47	Degrading β-catenin mRNA	Promoting TAM-based phagocytosis and glioma elimination	Tumour suppressor	(171)
	NSUN5		CTNNB1 caRNA	RBFOX2	Recruiting TET2, converting m5C to 5hmC degrading 5hmC caRNA	Promoting TAM-based phagocytosis and glioma elimination	Tumour suppressor	(171)
	NSUN5	Elevated	rRNA		Enhancing synthesis of oncoproteins	Potential tumourigenic risk	Tumour promoter	(156)
m7G	METTL1/WDR4	Elevated	tRNA	MAPK signalling pathway		glioma growth and proliferation	Tumour promoter	(110)
	METTL1/WDR4	Elevated	tRNA	SPP1 and PTN signalling pathways	Regulating TIMEs and ASEs		Tumour promoter	(154)
	RNMT/RAM	Elevated	mRNA	B7-H6/c-myc		Proliferation of GSCs	Tumour promoter	(81)
	WBSCR22	Elevated	rRNA	PI3K/AKT/GSK3β pathways		Proliferation and growth of glioma cells	Tumour promoter	(119)
m1A	TRMT6	Elevated	tRNA	PI3K-AKT, TGF-β, MTORC1, NOTCH, and MYC pathways	Regulating cell cycle	Proliferation, migration, and invasion of glioma cells	Tumour promoter	(112)

### 3.1 Role of RNA methylation in glioma tumorigenesis

**Figure 3:**
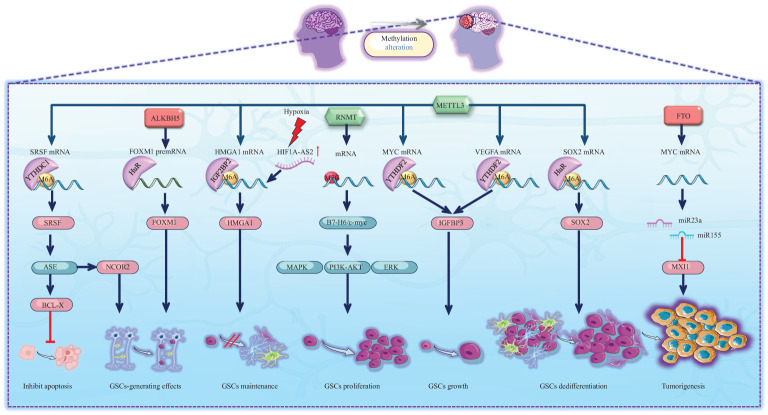
Schematic representation of RNA methylation alteration and its corresponding biological effects in tumourigenesis. Green icons represent writers (METTL3, RNMT), red icons represent erasers (ALKBH5, FTO). They determine the methylation status of RNAs, which is recognized by specific readers, leading to alterations in molecular expression levels and subsequent activation or inhibition of downstream pathways. The activities of METTL3, RNMT, ALKBH5, and FTO significantly regulate the biological behavior of GSCs, inhibiting their apoptosis and facilitating their generation, maintenance, proliferation, growth, and dedifferentiation, which ultimately contributes to tumour formation.

Glioma stem-like cells (GSCs) are crucial constituents within glioma tissues, contributing to the heterogeneity of gliomas and exerting a significant influence on glioma tumorigenesis, recurrence, and drug resistance (76). RNA methylation can significantly alter the biological behaviour of GSCs and thus contribute to the tumorigenesis of glioma (**Figure 3**). One study reported that METTL3, an m6A writer, methylates the SRY-box transcription factor 2 (SOX2) mRNA, which is recognized by human antigen R (HuR). Consequently, methylation increases SOX2 expression (13). Interestingly, with the addition of four developmental transcription factors (SOX2, OLIG2, SALL2, and POU3F2), differentiated glioma cells can be induced to differentiate into undifferentiated GSCs (77). Furthermore, METTL3 expression is increased in undifferentiated GSCs and attenuated during differentiation. These reports suggest that high METTL3 expression has a carcinogenic effect (77). Some bioinformatics studies have also revealed that high expression of METTL3 in glioma tissues can activate multiple oncogenic pathways and upregulate the expression of oncogenic factors, including those in the Notch pathway and NOTCH3, thereby promoting the onset of gliomas (73). Interestingly, another m6A writer, METTL14, together with METTL3, can inhibit ADAM19 mRNA and thus inhibit the growth and self-renewal of GSCs. Additionally, the expression of ADAM19 is increased after METTL14 knockdown, which promotes the occurrence of glioma (78). Depletion of METTL14 in the mouse embryonic nervous system also prolongs radial glial cell cycle progression and extends cortical neurogenesis to the postnatal stage (25). Intriguingly, the levels of m6A are relatively low in mouse brain tissue during embryogenesis but drastically increase in adulthood. A previous study demonstrated that m6A modification is regulated in a tissue-specific manner and is markedly increased throughout brain development (67). We conclude that METTL14 collaborates with METTL3 to form MTCs and that the role of the METTL14 subunit alone is more concentrated during neurogenesis.

In addition to the oncogenic effect of MTC, several m6A erasers also promote the occurrence of gliomas. For example, ALKBH5, an m6A eraser, demethylates the FOXM1 premRNA that can also be read by HuR, which increases pre-mRNA stability and thus enhances FOXM1 expression, leading to the formation of GSCs (79). Another m6A eraser, FTO, has been shown to promote glioma onset by targeting MYC mRNA. Mechanistically, FTO promotes the expression of the miR155 and miR23a cluster, thereby suppressing MXI1 expression. Eventually, FTO enhances tumorigenesis in U87 glioma cells (80). Notably, the MYC pathway is crucial for the onset of gliomas.

m6A methylation is commonly recognized as the primary mRNA methylation process. However, the onset of gliomas also involves various other types of RNA methylation-associated proteins. Researchers have reported that the m7G writer RNMT is involved in the B7-H6/c-myc axis. B7-H6 can activate the oncogene Myc and the PI3K/AKT and ERK/MAPK signalling pathways, leading to the proliferation of GSCs (81). Additionally, as previously mentioned, YTHDF2 is a reader that commonly promotes RNA degradation (25). Nevertheless, it is particularly noteworthy that YTHDF2 in GSCs plays a role in stabilizing MYC and VEGFA transcripts. IGFBP3 is a downstream factor of the YTHDF2-MYC axis. Along with high expression of YTHDF2, highly expressed IGFBP3 sustains GSC growth (82). In contrast, YTHDC1 is a reader that inhibits RNA degradation. METTL3 methylates the SRSF mRNA start codon, which can recruit YTHDC1 and inhibit nonsense-mediated mRNA decay (NMD). Ultimately, SRSF protein expression increases, leading to greater changes in alternative splicing events (ASEs), including BCL-X and NCOR2 transcript variant changes, which account for the antiapoptotic and GSC-generating effects, respectively. These effects eventually lead to GBM tumour outgrowth and self-renewal (83). In addition to altering ASEs, SRSFs themselves can promote methylation. SRSF7 methylates target mRNAs by recruiting MTC and is further recognized by IGF2BP2, thus stabilizing target mRNA (84). Although methylation promotes the variable splicing activity of SRSF, SRSF can in turn recruit MTC to change the methylation level of mRNA.

In addition to mRNA methylation, noncoding RNA methylation plays an important role in the oncogenesis of glioma. The let-7 miRNA is capable of differentiating cells by repressing stem cell programs. However, IGF2BP2, an m6A reader, binds to let-7 miRNA, thereby sustaining glioma cells in an undifferentiated state and maintaining GSCs, thus promoting the progression of glioma (85). Furthermore, IGF2BP2 is associated with the oxidative phosphorylation process. IGF2BP2 interacts with multiple mRNAs encoding subunits of the mitochondrial respiratory chain complex, thereby increasing the stability of components involved in oxidative respiration and promoting efficient oxidative phosphorylation in GSCs. Repression of oxidative phosphorylation leads to a decrease in the activity of GSCs, impeding the formation of cancer cell colonies (86). Interestingly, hypoxia promotes the expression of IGF2BP2 in GSCs by inducing the expression of hypoxia-inducible factor 1 alpha-antisense RNA 2 (HIF1A-AS2). Consequently, highly expressed IGF2BP2 stabilizes HMGA1 mRNA and contributes to the maintenance of GSCs (87). These findings indicate that RNA methylation plays a crucial role in the biological activity of GSCs through its impact on the oxidative phosphorylation chain. RNA methylation is involved in stabilizing components of the oxidative phosphorylation chain, thus stabilizing the metabolism of GSCs. However, under hypoxic conditions, GSCs can compensate for the detrimental effects of hypoxia by promoting high expression of IGF2BP2. Other studies have demonstrated that the expression of the lncRNA LINC00689 is upregulated in glioma, leading to increased expression of IGF2BP1 via the inhibition of miR-526b-3p. IGF2BP1 is an mRNA stabilizer for a series of oncogene-related pathways such as the MAPK signalling pathway (88, 89). In addition, some circRNAs are highly expressed in glioma, such as circNEIL3 (90) and circHIPK3 (91), which increase IGF2BP3 expression. Similar to IGF2BP1, IGF2BP3 is a carcinogenic protein that significantly promotes the progression of glioma. Furthermore, hnRNPC, another m6A reader, is highly expressed within the GBM microenvironment. This expression hinders the tumour immune microenvironment (TIME) via the immune checkpoint protein (ICP) network and activates the GALECTIN signalling pathway. Consequently, it significantly facilitates the stemness state in GBM cancer cells (92). Moreover, statistical analysis revealed that the upregulated expression of YTHDC2 is a risk factor for the occurrence of low-grade glioma (26). However, the specific carcinogenic mechanism is still worthy of further research in the future.

In conclusion, GSCs constitute integral components of glioma tissue. During the initiation of GSCs, substantial alterations in the methylation patterns of both mRNAs and noncoding RNAs occur. Predominantly, there are variations in m6A methylation accompanied by changes in m7G methylation levels. Ultimately, these shifts in methylation patterns are closely linked with multiple oncogenic pathways, significantly influencing the biological functions of GSCs and thereby promoting the initiation of gliomas (**Figure 3**).

### 3.2 Role of RNA methylation in glioma malignant progression, proliferation, invasiveness, and angiogenesis

**Figure 4:**
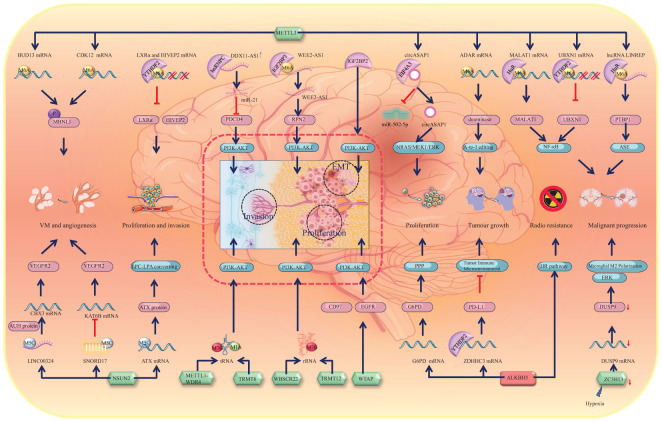
Schematic representation of RNA methylation alteration and its corresponding biological effects in glioma progression. Green icons represent writers (METTL3, NSUN2, METTL1-WDR4, TRMT6, WBSCR22, TRMT12, WTAP, and ZC3H13), red icons represent erasers (ALKBH5). They determine the methylation status of RNAs, which is recognized by specific readers, leading to alterations in molecular expression levels and subsequent activation or inhibition of downstream pathways. The dashed line box highlights the activation of the PI3K-AKT pathway, a core mechanism driving glioma progression, which promotes the EMT, proliferation, and invasion of glioma cells. Alterations in other molecules, such as upregulation of MBNL1 phosphorylation and VEGFR2 expression, contribute to glioma angiogenesis and vascular mimicry. Downregulation of LXRα and HIVEP2 enhances glioma invasion. Activation of other pathways, including NF-κB and ERK, drives glioma malignancy. The HR pathway activation promotes glioma radioresistance. Activation of NRAS, MEK1, ERK, and PPP supports glioma cell proliferation. RNA editing, such as A-to-I editing and ASE, is altered, which contributes to the malignant progression of glioma.

In addition to contributing to the oncogenesis of glioma, methylation of mRNAs leads to the activation of a series of pathways that significantly contribute to the progression of malignant gliomas (14, 93). To comprehensively understand the underlying mechanisms of RNA methylation in the progression of gliomas, we have provided an overview of the involvement of RNA methylation and its associated proteins in various aspects of glioma progression, including proliferation and growth, invasiveness, epithelial-mesenchymal transition (EMT), and angiogenesis (**Figure 4**).

The changes in RNA m6A methylation levels represent a primary mechanism underlying the formation of GSCs and the onset of gliomas. Similarly, significant alterations in RNA methylations also occur during glioma progression, with the role of m6A methylation in promoting glioma progression being extensively studied and mechanistically well understood. METTL3, an m6A writer, methylates the mRNAs of MALAT1 and UBXN1, which are recognized by the m6A readers HuR and YTHDF2, respectively. Consequently, HuR stabilizes MALAT1 mRNA and promotes its expression, whereas YTHDF2 mediates UBXN1 mRNA degradation and decreases its expression. Consequently, both pathways activate NF-κB signalling and promote the malignant progression of gliomas (14, 74). Furthermore, NF-κB signalling can also be activated by the m6A reader EIF3i, which can affect mRNA processing, translation, and TCR signalling and is associated with the malignancy of glioma (93). Thus, the NF-κB signalling pathway has emerged as a pivotal pathway driving the malignant progression of gliomas.

In addition to its role in malignant progression, studies have revealed that METTL3 increases astrocyte immortality by recruiting RasV12, which accounts for the growth of glioma tumours (13). Furthermore, METTL3 methylates and stabilizes the adenosine deaminase acting on RNA (ADAR) mRNA, which encodes a deaminase with mRNA binding activity. Consequently, increasingly expressed ADAR1 binds to CDK2 mRNA and activates A-to-I editing, which significantly promotes glioma growth (94). Furthermore, the co-upregulation of METTL3 and ALKBH5 promotes the activation of the homologous recombination (HR) pathway. This reduces the radiosensitivity of glioma tissues, but it also significantly enhances their invasiveness (13, 95). The demethylase ALKBH5 has also been reported to facilitate the growth of gliomas. The downregulation of ALKBH5 expression promotes the recognition and degradation of ZDHHC3 mRNA by YTHDF2. Inhibition of ZDHHC3 mRNA expression can reduce the expression of the PD-L1 protein, thereby activating tumour immunity and disrupting the suppressive TIME, ultimately inhibiting glioma growth (96). Additionally, ALKBH5 can also impact the progression of gliomas by influencing the oxidative phosphorylation chain. ALKBH5 demethylates the G6PD transcript and enhances its mRNA stability, thereby promoting G6PD translation and activating the pentose phosphate pathway (PPP), consequently enhancing glioma cell proliferation (97). Interestingly, the chaotic progression of malignant tumours leads to the formation of localized hypoxic conditions within and around the tumour, which leads to the release of miR-200c-3p from neurons to the surrounding area and the activation of local neurons. Consequently, the expression of ZC3H13 decreases in microglia and inhibits the methylation of dual specificity phosphatase 9 (DUSP9) mRNA. Downregulation of DUSP9 promotes ERK pathway activation, resulting in microglial M2 polarization, which promotes the progression of glioma (98). Similarly, owing to the activation of the EGFR/SRC/ERK pathway, YTHDF2, an m6A reader, is highly expressed. The highly expressed YTHDF2 facilitates m6A-dependent mRNA decay of LXRα and HIVEP2, which promotes GBM cell proliferation and invasion (99). NSUN2 methylates the 3’-UTR of ATX mRNA, which increases the expression of the ATX protein, converting lysophosphatidylcholine (LPC) into lysophosphatidic acid (LPA). Consequently, this alteration further promotes the migration and proliferation of glioma cells (33). Moreover, it is important to highlight that TRIM29 is a factor intricately linked to inflammation. It plays a role in modulating endoplasmic reticulum (ER) stress, apoptosis, reactive oxygen species (ROS) responses, and inflammasome activation via the protein kinase RNA-like endoplasmic reticulum kinase (PERK) and other pathways (100, 101). In the context of glioma initiation and progression, TRIM29 serves as a significant immunosuppressive factor. Mechanistically, via degrading the adaptor NEMO, TRIM29 inhibited interferon-regulatory factors and NF-κB signaling, as it directly interacted with NEMO and triggered its ubiquitination and breakdown (102–104). Throughout glioma progression, the recruitment of YTHDF1 facilitates an elevation in m6A-modified TRIM29 mRNA levels, subsequently leading to the upregulation of tumor stem cell markers CD44 and CD133 (105). The upregulation observed in gliomas facilitates tumor growth and invasion via the activation of the Wnt/β-catenin signaling pathway (106, 107).

In addition to mRNAs, methylation-associated proteins can also modify noncoding RNAs, thereby contributing to the progression of gliomas. METTL3 is involved in the m6A methylation of the lncRNA LINREP and maintains its stability by recruiting HuR. Consequently, highly expressed LINREP confers protection upon polypyrimidine tract-binding protein 1 (PTBP1) against degradation, thereby significantly impeding ASEs and promoting glioma progression (108). Previous studies have demonstrated that the m6A reader EIF4A3 interacts with the circASAP1 flanking sequence, leading to upregulation of its expression. This upregulation subsequently activates the NRAS/MEK1/ERK1–2 signalling pathway by sequestering miR-502–5p, thereby promoting glioma cell proliferation (109). The function of tRNA can also be affected by RNA methylation. The METTL1-WDR4 complex participates in the m7G modification of tRNA and prevents its degradation. Researchers have reported that aberrant expression of METTL1 is associated with brain malformation and multiple malignancies (46). Interestingly, METTL1 is highly expressed in glioma and significantly promotes its growth and proliferation via activation of the MAPK pathway (110). In addition to METTL1, TRMT6 and TRMT61 can promote malignant transformation and progression by sustaining tRNA methylation in glioma (111). Bioinformatics screening revealed that TRMT6 is highly expressed in high-grade gliomas and is involved in regulating the cell cycle and the PI3K-AKT, TGF-β, MTORC1, NOTCH, and MYC pathways to promote glioma proliferation. The inhibition of TRMT6 significantly suppresses the proliferation, migration, and invasion of glioma cells (112). Within this cascade of pathways, the PI3K-AKT pathway can also be activated by elevated IGF2BP2 in gliomas, thereby promoting GBM cell proliferation, migration, invasion, and EMT (113). In fact, the PI3K-AKT pathway plays a particularly crucial role in the progression of gliomas, with various RNA methylations promoting glioma progression by influencing this pathway. For example, WTAP is a nuclear protein that regulates the activity of epidermal growth factor receptor (EGFR) and has been reported to be associated with cell proliferation and apoptosis (114). WTAP activates the PI3K-AKT and extracellular signal-related kinase pathways, thereby promoting glioma cell proliferation, migration, and invasion (115). WTAP is highly expressed in glioma, and its expression is closely correlated with glioma grade. Interestingly, the gene encoding WTAP (WT1) is closely related to CD97. CD97 is also a member of the EGFR family. Increased CD97 expression promotes cellular invasiveness and tumour migration (116–118). WBSCR22 and TRMT112 are responsible for mediating m7G methylation in rRNA (47). A recent study demonstrated that WBSCR22 is upregulated in glioma and increases its growth and proliferation via activation of the PI3K/AKT/GSK3β pathways (119). The m6A writer hnRNPC promotes miR-21 expression in T98G cells. Interestingly, the combination of miR-21 with programmed cell death 4 (PDCD4) can inhibit PDCD4 expression, which inhibits apoptosis and promotes glioma invasive activities via the promotion of AKT and p70S6K pathway activation (120). Moreover, the expression of the long noncoding RNA DDX11 antisense RNA 1 (DDX11-AS1) is increased in gliomas. DDX11-AS1 interacts with hnRNPC and thus activates the Wnt/β-catenin and AKT pathways, which promote the EMT process in gliomas (121).

In general, the NF-κB signalling pathway plays a crucial role in the malignant progression of gliomas, whereas the PI3K-AKT pathway contributes to glioma proliferation and growth and facilitates glioma invasiveness and migration (**Figure 4**).

In addition to participating in the proliferation and growth of gliomas, METTL3 is involved in the formation of the glioma vasculature. It stabilizes the mRNAs of BUD13 and CDK12, thereby promoting MBNL1 phosphorylation and subsequently facilitating the formation of glioma vasculogenic mimicry (VM) (122). Notably, however, the downregulation of METTL3 expression and the upregulation of expression of the eraser ALKBH5 can also promote VM (123). This paradoxical phenomenon requires further investigation. The m5C writer NSUN2 is also increased in glioblastoma endothelial cells, which increases the stability of LINC00324 by m5C modification and upregulates its expression. The highly expressed LINC00324 binds with the AUH protein. Thus, the ability of the AUH protein to bind to CBX3 mRNA is reduced. LINC00324 thus increases the expression of CBX3, which binds to the promoter region of VEGFR2 to promote the expression of VEGFR2, promoting angiogenesis in glioma (124). Similar to LINC00324, many noncoding RNAs, such as the small nucleolar RNA SNORD17, are highly expressed in glioma tissues. SNORD17 can promote the methylation of KAT6B mRNA, leading to decreased KAT6B expression, which in turn promotes the upregulation of VEGFR2 and VE-cadherin expression, thereby facilitating VM in GBM (125).

Given that RNA methylation-associated proteins promote glioma development, reversing this trend could inhibit glioma progression. We found that EIF3b knockdown can inhibit the proliferation of glioma U87 cells through cell cycle arrest in the G0-G1 phase and promote apoptosis (126). Another report demonstrated that hnRNPA2/B1 knockdown leads to inactivation of the AKT and STAT3 signalling pathways, which ultimately reduces the expression of B-cell lymphoma-2 (Bcl-2), cyclin D1, and proliferating cell nuclear antigen (PCNA) (127). The hnRNPA2B1 ablation exhibited a significant tumour-suppressive effect on glioma cell proliferation, GSC self-renewal and tumorigenesis (128). YB-1 is an m5C reader that affects the expression of key proteins and the phosphorylation of key pathways involved in the cell cycle, adhesion, and apoptosis in gliomas (129). It is highly expressed in glioma tissues. The upregulated YB-1 protein binds to CCT4 mRNA to promote its expression, which enhances the mLST8 folding process and eventually activates the mTOR signalling pathway in glioma (130). YB-1 is also highly expressed in glomeruloid microvascular endothelial cells of brain tumours and microvessels in the desmoplastic region around multiple solid tumours. Inhibition of this high expression can decrease the vasogenic effect of growth factors, thus inhibiting tumour growth (131).

In summary, significant alterations in the methylation of mRNAs and noncoding RNAs occur during the malignant progression of gliomas. The majority of alterations in mRNA methylation directly contribute to glioma progression by activating specific signalling pathways. For instance, the activation of the NF-κB signalling pathway is implicated in the malignant advancement of gliomas, while the PI3K-AKT pathway is known to enhance glioma proliferation and growth, as well as facilitate invasiveness and migration. Nevertheless, some of these pathways exhibit correlational relationships, and the mechanisms leading to glioma progression are multifaceted. For example, the m5C reader influences the expression of key proteins and the phosphorylation of critical pathways involved in the cell cycle, adhesion, and apoptosis in gliomas (129). In conclusion, we have systematically reviewed the complete signalling pathways currently reported to be associated with RNA methylation and glioma progression. Among these modifications, the most important is m6A modification. Additionally, m7G, m5C, and m1A also exhibit varying degrees of alteration. These alterations in methylation levels ultimately activate downstream pathways that drive a sequence of malignant progression events in gliomas. These processes include proliferation, growth, invasion, EMT, and angiogenesis. m6A modification changes across these progression stages and broadly impacts the methylation levels of both mRNAs and noncoding RNAs. Intriguingly, the m7G, m5C, and m1A modifications influence the methylation status of noncoding RNAs. The effects of m6A, m7G, m5C, and m1A methylation are intricately regulated by a network involving writers, erasers, and readers, thereby contributing to the evolution of glioma cell biological behaviours.

### 3.3 Role of RNA methylation in glioma drug resistance

Drug resistance in gliomas is one of the primary reasons for their high degree of malignancy, low survival rates, and propensity for recurrence. Previous studies have revealed the critical role of RNA methylation alterations in the development of drug resistance in gliomas. Therefore, gaining a deeper understanding of the mechanisms by which RNA methylation leads to glioma drug resistance is imperative.

Temozolomide (TMZ) is the most common and fundamental chemotherapeutic drug used to treat malignant gliomas (132, 133). Research has revealed a close correlation between the methylation levels of RNA and the emergence of resistance to TMZ. In 2008, it was first concluded that MGMT promoter methylation is a recognized test indicator for predicting the efficacy of TMZ in the treatment of glioma (75). Therefore, we examine the specific mechanisms by which RNA methylation leads to resistance to TMZ (**Table 1**).

m6A methylation is the most prevalent RNA modification observed to be significantly altered in gliomas. Interestingly, the m6A writer METTL3 stabilizes the mRNAs of MGMT and ANPG, which are two molecules responsible for critical DNA repair processes. Consequently, high METTL3 expression results in increased resistance to TMZ in GSCs (134, 135). The findings of these studies pave the way for the proposition of broadly reducing methylation to increase glioblastoma TMZ sensitivity (134). In addition to m6A writers, some erasers and readers can also promote TMZ resistance in gliomas. For example, the m6A reader EIF4 has been shown to increase glioma drug resistance. EIF4A3 activates the NRAS/MEK1/ERK1–2 signalling pathway by upregulating the expression of circASAP1, which significantly promotes glioma reproduction and TMZ resistance (109). Moreover, the m6A eraser FTO was reported to reduce the efficacy of TMZ by targeting the MYC signalling pathway (80). Additionally, elevated circ_0072083 inhibits the expression of miR-1252-5p, resulting in increased ALKBH5 levels that contribute to the demethylation of Nanog homeobox (NANOG) mRNA. This increase in NANOG expression also results in increased TMZ resistance in gliomas (136). Interestingly, ALKBH5 can also enhance glioma resistance to TMZ by stimulating the expression of SOX2, which coincides with the role of METTL3 in stabilizing GSCs through the promotion of SOX2 expression. ALKBH5 demethylates SOX2 mRNA, consequently stabilizing it and leading to SOX2 overexpression, which in turn activates the Wnt5α/β-catenin signalling pathway. This not only promotes glioma cell proliferation but also enhances TMZ resistance (137). Moreover, the upregulation of SOX2 mediated by METTL3 and the increased expression of ALKBH5 also enhance the radioresistance of glioma. Both contribute to activation of the HR pathway, thereby promoting DNA damage repair and radioresistance (95). In summary, increased expression of ALKBH5 poses a significant obstacle to glioma therapy. Further elucidating its pathogenic mechanisms and investigating strategies to inhibit the effects of ALKBH5 will be important research directions for the future. ZC3H13, which acts as a stabilizer of the MTC, facilitates the localization of the ZC3H13-WTAP-Virilizer-Hakai complex in the nucleus, thereby maintaining normal RNA m6A methylation (19, 20). Researchers have revealed that the ZC3H13 mutation (decrease) significantly disrupts the balance of intracellular methylation, which increases the resistance of glioma to TMZ (138).

In addition to conferring TMZ resistance, RNA methylation-related proteins also contribute to resistance against other targeted drugs, thereby posing significant challenges in the treatment of gliomas. For example, in the glioblastoma U87 cell line, YTHDF3 can suppress the expression of p21 by inhibiting the p21 signalling pathway, thereby promoting resistance to a targeted drug, osimertinib (139). Furthermore, hnRNPA1 binds methylated cyclin D1 and c-myc internal ribosome entry site (IRES) mRNAs, leading to elevated IRES activity and drug resistance protein synthesis, which is correlated with increased resistance of GBM to mTOR inhibitors (140). In most reported cases, METTL3 promotes the occurrence of gliomas. Studies have reported that high METTL3 expression in gliomas increases their resistance to antiangiogenic drugs. The specific mechanism involves METTL3 stabilizing the mRNAs of BUD13 and CDK12, leading to their overexpression, which in turn promotes the phosphorylation of MBNL1 and facilitates the VM process in gliomas (122).

In summary, METTL3-mediated m6A methylations represent crucial factors contributing to drug resistance in gliomas. These methylation alterations can induce resistance to the conventional chemotherapy drug TMZ and interfere with other targeted therapies. Therefore, given that the MGMT promoter methylation level can be used to predict the curative effects of TMZ (75), combining chemotherapy with drugs that target methylation changes may represent a promising new therapeutic approach for treating gliomas in the future.

### 3.4 RNA methylation-associated proteins inhibit the progression of gliomas

In most cases, increased levels of RNA methylation and the expression of associated proteins contribute to the deterioration of gliomas, including their initiation, progression, and resistance to drug therapy. Nevertheless, several studies have demonstrated that certain methylation-related proteins can inhibit the progression of gliomas and may serve as protective factors. These proteins, such as FTO, are demethylases that reverse the high methylation levels typically found in gliomas. Although FTO is upregulated in gliomas, it may counteract abnormal methylation, serving as a compensatory mechanism that reduces the malignancy of gliomas. However, some methyltransferases also exhibit antiglioma effects. This might occur because methyltransferases stabilize the disrupted methylation network in glioma tissue or because methyltransferases can recruit demethylases to maintain cellular methylation homeostasis.

Although reports suggest that FTO can promote the onset of gliomas (80), notably, in most cases, the expression of FTO in gliomas is significantly negatively correlated with tumour malignancy. Therefore, FTO is generally considered a protective factor against glioma progression. Furthermore, reports indicate that FTO can inhibit glioma growth and invasion, making it a potential suppressor of GBM (141). Mechanistically, FTO promotes the nuclear translocation of FOXO3a, resulting in high expression of FOXO3a downstream factors such as BIM, BNIP3, BCL-6, and PUMA. This inhibits the hypoxia-induced capacity of glioma cells to proliferate, migrate and invade (98, 141). Artificially blocking the high expression of FTO via the application of miR-27a-3p could significantly promote the progression of glioma (141). Similarly, the downregulated expression of FTO enhances the m6A modification of primary microRNA-10a (pri-miR-10a), which can be recognized by the reader HNRNPA2/B1 and increase the glioma tumour burden (142).

Although many studies have reported that METTL3 promotes the occurrence and progression of gliomas (13, 14, 74, 77), METTL3 also has inhibitory effects on glioma occurrence. Highly expressed METTL3 reduces the mRNA level of a disintegrin and metallopeptidase domain 19 (ADAM19). Consequently, it inhibits the growth and self-renewal of GSCs. After METTL3 knockdown, ADAM19 expression increases, which promotes the occurrence of glioma (78). Moreover, silencing METTL3 also results in downregulated expression of ADAR while increasing the expression of apolipoprotein B mRNA-editing enzyme catalytic subunit 3A (APOBEC). This alteration leads to a series of aberrant ASEs, specifically, the A-to-I and C-to-U RNA editing events in GSCs, and eventually activates oncogenic pathways associated with the development of gliomas (143). In addition to mRNAs, METTL3 mediates the methylation of circRNAs, which methylates and stabilizes circDLC1. This process enhances CTNNBIP1 expression via competitive binding with miR-671-5p. Consequently, highly expressed CTNNBIP1 significantly inhibits glioma cell proliferation (144). Another study reported that METTL3 expression is decreased in the U251 glioma cell line. Along with increased expression of FTO, the m6A modification level significantly decreases and consequently increases the expression of HSP90, which interferes with the apoptotic system and promotes glioma development (145).

In conclusion, the regulation of tumour progression by RNA methylation constitutes a complex network in which both excessively high and low levels of RNA methylation can potentially promote the occurrence and progression of gliomas (**Table 1**). Therefore, maintaining the homeostasis of RNA methylation within cells is crucial for normal function and provides a novel perspective for glioma therapies.

## 4 Potential diagnostic implications of RNA methylation in GBM

A 2004 study reported that the DNA m5C modification can be used as a predictive biomarker for the diagnosis and recurrence of gliomas (146). Recently, a scoring system based on the level of RNA m6A methylation was proposed. Patients with low m6A methylation scores tend to exhibit normal T-cell functionality in the tumour microenvironment in contrast to those with high m6A methylation scores. Moreover, the former patients also show better treatment responsiveness to chemotherapy drugs such as bevacizumab and regorafenib (147). These findings shed light on the broad prospects of utilizing RNA methylation for diagnosing gliomas. An increasing number of studies have reported that with the development of glioma, the RNA methylation level changes significantly. Although the specific molecular mechanisms of glioma carcinogenesis remain to be further explored, alterations in RNA methylation during glioma development can be detected manually, which makes it possible to detect glioma earlier and better predict its prognosis. We summarize the trends of alterations in RNA methylation-related molecules in glioma tissues during the course of the disease, and also summarize several possible biomarkers for glioma diagnosis and prognosis (**Table 2**).

**Table 2:** Prognostic value of RNA methylation-associated proteins in gliomas.

Methylation types	Roles in RNA Modification	RNA methylation regulators	Expression in gliomas	Prognostic value in gliomas	Refs
m6A	m6A Writers	METTL3	Controversial	Mainly higher malignant grade and poorer prognosis of IDH-wildtype gliomas; Secondarily longer median overall survival (OS) time	(13, 14, 123)
	m6A Writers	METTL14	Elevated	Protective factor	(78)
	m6A Writers	WTAP	Elevated	Poor prognosis	(116)
	m6A Writers	KIAA1429	To be confirmed	To be confirmed	
	m6A Writers	RBM15/15B	Elevated	IDH-wildtype GBM	(148, 149)
	m6A Writers	ZC3H13	Decreased	Increasing TMZ resistance	(138, 148)
	m6A Erasers	FTO	Controversial	Lower expression implies higher glioma grades and poorer clinical outcomes	(80, 142, 148)
	m6A Erasers	ALKBH5	Elevated	Shorter median overall survival (OS) time	(123)
	m6A Readers	YTHDF1	Elevated	Risk factors for HGG poor prognosis and chemoresistance	(151, 152)
	m6A Readers	YTHDF2	Elevated	Risk factors for HGG poor prognosis	(74, 99)
	m6A Readers	YTHDF3	Elevated	Risk factors for HGG	(56)
	m6A Readers	YTHDC1	Elevated	Protective factors for HGG	(56)
	m6A Readers	YTHDC2	Elevated	Risk factors for LGG onset and poor prognosis	(26)
	m6A Readers	IGF2BP1	Elevated	Poor prognosis	(88, 89)
	m6A Readers	IGF2BP2	Elevated	TMZ resistance	(113)
	m6A Readers	IGF2BP3	Elevated	Poor prognosis	(90, 91)
	m6A Readers	HNRNPC	Elevated	Better prognosis	(150)
	m6A Readers	HNRNPA2B1	Elevated	To be confirmed	(148)
	m6A Readers	EIF3i	Elevated	Independent prognostic factor for poor prognosis of IDH-mutant LGG	(93)
m5C	m5C Writers	NSUN2	Elevated	Independent biomarker for prognostic evaluation in patients with LGG	(155)
	m5C Writers	NSUN5	Elevated	Protective factor but requires further investigation	(171)
	m5C Writers	Other NSUN	To be confirmed	To be confirmed	
	m5C Erasers	TET1	To be confirmed	Protective factor	(36)
	m5C Erasers	TET2	To be confirmed	Protective factor	(36)
	m5C Erasers	TET3	To be confirmed	Protective factor	(36)
	m5C Readers	YB-1	Elevated	Shorter median overall survival (OS) time	(129)
	m5C Readers	ALYREF	Elevated	LGG poor prognosis	(155, 158)
m7G	m7G Writers	METTL1	Elevated	Poor prognosis	(110, 154)
	m7G Writers	WDR4	Elevated	Poor prognosis	(110, 154)
	m7G Writers	WBSCR22	Elevated	Poor prognosis	(119)
	m7G Writers	TRMT112	To be confirmed	To be confirmed	
	m7G Writers	RNMT	Elevated	Risk factor	(81)
	m7G Writers	RAM	Elevated	Risk factor	(81)
m1A	m1A Writers	TRMT6	Elevated	Poor prognosis	(111, 112)
	m1A Writers	TRMT61	Elevated	Poor prognosis	(111, 112)
	m1A Erasers	ALKBH1	Elevated	Risk factors for HGG	(56)
	m1A Erasers	ALKBH3	Elevated	Risk factors for HGG	(56)

### 4.1 RNA m6A methylation levels and associated proteins serve as diagnostic and prognostic biomarkers

METTL3 is one of the most important m6A writers of RNA. We have reported that high METTL3 expression in GSC-rich GBM is indicative of a poor prognosis (13). Moreover, METTL3 expression is positively associated with a higher malignant grade and poorer prognosis in IDH-wild-type gliomas but not in IDH-mutant gliomas (14). As one of the substrates of METTL3, high expression of SRSF7 also significantly promotes the progression of glioma (84). However, completely contradictory results have been reported, revealing that patients with relatively high expression of METTL3 have prolonged disease-free survival (123). Thus, the diagnostic value of METTL3 is debatable and requires further investigation. WTAP, another important component of the m6A writer, has increased expression in glioma and can predict poor postoperative survival in glioma patients. Thus, WTAP may serve as a good novel prognostic biomarker (116). In addition to WTAP, the expression of METTL14, RBM15, and its paralogue RBM15B is also increased in gliomas (148). It has been reported that increased expression of RBM15 has prognostic value in IDH-wildtype GBM (149). However, not all m6A writer components are elevated during the development of glioma. The expression of ZC3H13 is generally decreased in glioma tissues (148), which increases the resistance of glioma to TMZ (138).

For m6A erasers, the expression of FTO is generally lower in glioma tissues than in normal tissues (148). Decreased FTO expression in clinical samples is correlated with higher glioma grades and poorer clinical outcomes (142). Interestingly, in contrast to FTO expression, increased ALKBH5 expression in gliomas implies a poorer prognosis. Patients with higher expression of ALKBH5 were reported to have a significantly shorter median overall survival (OS) time (123). Furthermore, depleting ALKBH5 expression is responsible for disrupting the tumorigenesis process of gliomas (79). Thus, increased expression of ALKBH5 and decreased expression of FTO can be used to predict the poor prognosis of gliomas.

With respect to m6A readers, according to a Lasso-Cox regression algorithm, glioma patients with high expression of HNRNPC had a good prognosis (150). These findings suggest that high HNRNPC expression can be used to predict a better prognosis in glioma patients. Nevertheless, the high expression of most other m6A readers implies a poor prognosis in gliomas. For example, the m6A reader YTHDF1 positively regulates GBM proliferation, chemoresistance, and cancer stem cell-like properties. Studies have confirmed that high expression of YTHDF1 predicts a poor prognosis in glioma patients (151, 152). In addition, another study demonstrated that YTHDF2 is overexpressed in glioma, which promotes its malignant progression both *in vitro* and *in vivo (* 74). Clinically, YTHDF2 overexpression is also correlated with poor glioma patient prognosis (99). K-M survival curve showed the prognostic significance of YTHDC2 expression in the context of LGG, which is significantly elevated in LGG (26). Other readers, such as IGF2BP3, can be regulated by circRNAs, specifically circNEIL3 and circHIPK3. The expression of circNEIL3 and circHIPK3 is upregulated in glioma tissues, and this elevated expression level is closely linked to poor patient prognosis (90, 91). Moreover, high expression of eIF3i could be used as an independent prognostic factor for poor prognosis in IDH-mutant LGG (93), which may be highly useful in the diagnosis and treatment of glioma. In addition, the expression levels of YTHDF3 and hnRNPA2/B1 are elevated in gliomas (148), but they may not have as much independent clinical significance as the highly expressed eIF3i in LGG.

In conclusion, among the currently known m6A readers, although highly expressed HNRNPC is a protective factor for glioma, high HNRNPC expression of other readers always indicates a poor prognosis in glioma patients. Expression of some of these readers can even serve as independent predictors of poor prognosis in patients with gliomas.

### 4.2 Other RNA methylation levels and associated proteins serve as diagnostic and prognostic biomarkers

Similar to the m6A methylation scoring system in glioma patients, which has been demonstrated to predict prognosis (147), a glioma m5C signature has also been established for prognosis prediction (153). Moreover, a predictive nomogram for the m7G modification score has been developed, and the prognostic value of detecting m7G modifications in gliomas has been explored (154). Additionally, recent studies have indicated that m1A-associated proteins can be used to assess the prognosis of gliomas. Collectively, these findings highlight the diagnostic and prognostic significance of m5C, m7G, and m1A methylation in gliomas.

The ability of the m5C writer to predict the prognosis of glioma patients is mainly related to its ability to predict the prognosis of LGG patients. The expression of NSUN2 was found to be upregulated, and this upregulation of NSUN2 serves as an independent biomarker for prognostic evaluation in patients with LGG (155). NSUN5 specifically participates in the methylation process of 28S rRNA, which promotes the synthesis of a series of carcinogenic proteins and thus plays a crucial protumorigenic role. Therefore, highly expressed NSUN5 can be utilized to predict the prognosis of gliomas (156). Moreover, the upregulation of m5C writers contributes to the high methylation of four lncRNAs, including LINC00265, CIRBP-AS1, GDNF-AS1, and ZBTB20-AS4. The m5C methylation level of these genes is associated with LGG prognosis. Therefore, monitoring the m5C methylation levels of these four RNAs can serve as a valuable prognostic tool for LGG, with high expression indicating an unfavourable outcome (157).

The m5C reader includes YB-1 and ALYREF, both of which are reported to have prognostic value. The overexpression of YB-1 has been demonstrated to be positively associated with glioma progression and inversely correlated with patient overall survival (OS) (129). Thus, YB-1 can be considered a risk factor for poor prognosis in glioma patients. Although the carcinogenic mechanism of ALYREF is unclear, it has been reported that high expression of ALYREF can be used to predict poor prognosis in patients with LGG (155). Moreover, increased expression of ALYREF has been reported in gliomas, which also verifies its prognostic value (158).

A predictive nomogram for the m7G modification score has been established that explores the prognostic value of detecting the m7G modality in gliomas. In addition, an analysis of the TCGA and GEO databases has revealed that transcriptomic alterations in genes associated with m7G methylation regulators are linked to the prognosis of gliomas (159). Mechanistically, excessive m7G methylation is positively associated with activation of the SPP1 and PTN signalling pathways. This results in changes in the TIMEs and ASEs, which are associated with the poor prognosis of gliomas (154). Among the m7G writers, METTL1 and WBSCR22, both of which are highly expressed in glioma tissues and promote their proliferation and growth, are closely associated with the prognosis of glioma patients (110, 119). Thus, increased expression of METTL1 and WBSCR22 may be used to determine the prognosis of glioma patients.

With respect to m1A methylation, inhibition of TRMT6 suppressed the proliferation, migration, and invasion of glioma cells. The elevated expression of TRMT6 may serve as a powerful and independent biomarker for poor prognosis in glioma (112). Moreover, a concomitant increase in TRMT6/TRMT61 mRNA and tRNAi (Met) expression with decreased expression of PKCα mRNA was detected in highly aggressive glioblastoma as compared with Grade 2/3 glioma (111). In conclusion, through the comparison of LGG and HGG, increased m1A modification markedly affected glioma prognosis. We conclude that the upregulation of TRMT6 and TRMT61 can be used to predict a poor prognosis. In addition, other studies have demonstrated that the overexpression of NML and TRMT10C can be considered risk factors for gliomas (56).

Generally, the RNA methylation scoring system provides a good research perspective and could serve as an auxiliary diagnostic tool for gliomas in the future. In addition, monitoring alterations in the expression levels of other RNA methylation-related proteins is crucial for the diagnosis and prognostic assessment of gliomas.

## 5 From bench to bedside: potential therapeutic implications of RNA methylation in gliomas

To date, multiple potential therapeutic targets have been identified based on RNA methylation (160). Generally, these therapeutic targets focus on the m6A methylation of RNA. Additionally, the modification of some noncoding RNAs has also become an increasingly important direction in the study of glioma therapy. We summarize the existing methods for treating gliomas by altering RNA methylation (**Table 3**).

**Table 3:** RNA methylation-associated protein-targeted treatments in gliomas.

Molecule (drugs)	Regulatory roles	Targets	Target roles in RNA Modification	Biological effects	Refs
DAA	Inhibitor	METTL3	m6A writer	Restoring TMZ sensitivity	(134)
miR-29a	Inhibitor	WTAP	m6A writer	Inhibiting cell proliferation, migration, and invasion but promoting apoptosis in GSCs	(115)
R-2HG	Inhibitor	FTO	m6A eraser	Disrupting the stability of oncogenic transcripts and inhibiting the activation of downstream oncogenic pathways	(165)
MA2	Inhibitor	FTO	m6A eraser	Enhancing the effect of TMZ on suppressing proliferation of glioma cells	(80)
DB2313	Promotor	FTO	m6A eraser	Decreasing GBM tumour burden	(142)
IOX1	Inhibitor	ALKBH5	m6A eraser	Enhancing the therapeutic efficacy of anti-PD-1 treatment	(96)
miR-1252-5p	Inhibitor	ALKBH5	m6A eraser	Enhancing the sensitivity of glioma tissues to TMZ	(136)
miR-526b-3p	Inhibitor	IGF2BP1	m6A reader	Reducing the incidence of gliomas	(88)
miR-4500	Inhibitor	IGF2BP1	m6A reader	Inhibit the development of gliomas	(166)
WEE2-AS1	Promotor	IGF2BP3	m6A reader	Blocking WEE2-AS1 expression improved the therapeutic sensitivity of dasatinib	(72)
circNEIL3/circHIPK3	Promotor	IGF2BP3	m6A reader	Decreasing the expression of circNEIL3 and circHIPK3 reduced glioma occurrence	(90, 91)
Musashi-1	Promotor	YTHDF1	m6A reader	Decreasing Musashi-1 reduced GSCs	(151)
Linsitinib	Inhibitor	IGF1/IGF1R YTHDF2-expressing cells	m6A reader	Inhibiting GSC viability, impairing glioblastoma growth	(82)
miR-129-5p	Inhibitor	DNMT3A	m5C writer	Suppressing the proliferation of glioma cells	(167)
YBX1-1/YBX1-2	Inhibitor	YB-1	m5C reader	Slower tumour growth	(130)
PKCα	Inhibitor	TRMT6/61	m1A writer	Inhibiting malignant transformation and progression of gliomas	(111)

### 5.1 Targeting m6A

As one of the most significant m6A writers, METTL3 has great potential for the development of drugs to treat gliomas. In the majority of cases, METTL3 is known to promote glioma development, and studies have reported that its overexpression enhances the formation of VM in gliomas, thereby increasing glioma resistance to treatment. Inhibiting the methylation of METTL3 or knocking down its downstream upregulated factors, such as BUD13 and CDK12 (122), can significantly suppress VM occurrence. This may become a pivotal strategy for future glioma therapies. It is worth noting that the upregulation of METTL3 and various m6A methylation levels significantly promote glioma cell resistance to TMZ. Nevertheless, an inspiring recent study revealed that DAA-mediated methylation blockade can restore TMZ sensitivity in GBM cells (134). These findings suggest that reducing overall methylation levels may contribute to maintaining methylation homeostasis within cells and provide novel insights into potential therapeutic methods for GBM. One study revealed that miR-29a inhibits mRNA expression of the Quaking gene isoform 6 (QKI-6) by binding to its 3’-UTR, which inhibits the expression of another m6A writer, WTAP. Consequently, downregulated WTAP inhibits the PI3K-AKT and extracellular signal-related kinase pathways, thereby inhibiting cell proliferation, migration, and invasion but promoting apoptosis in GSCs. Thus, miR-29a can be used as a potential therapeutic agent (115).

In addition to targeting m6A writers, studies have reported that m6A erasers may serve as promising therapeutic targets for glioma. IDH mutations block cell differentiation and promote tumour transformation, and the inhibition of mutant IDH (IDHi) can reverse this effect. Thus, as the major metabolic product of IDH mutants, R-2HG has been regarded as an oncometabolite (161–164). However, R-2HG inhibits FTO in glioma, causing MYC/CEBPA mRNA methylation, disrupting the stability of these oncogene transcripts and inhibiting the activation of downstream oncogene pathways. Thus, R-2HG has an antiglioma effect (165). This explains why the IDH mutation is deemed a benign mutation in gliomas and could be used to predict a better prognosis. Meclofenamic acid (MA2) also inhibits the expression of FTO, thus enhancing the ability of the chemotherapy drug TMZ to suppress the proliferation of glioma cells (80). Therefore, inhibiting FTO overexpression may be an effective method for treating glioma. However, another study reported contradictory results. Researchers have shown that application of the SPI1 inhibitor DB2313 increases FTO expression and decreases the GBM tumour burden (142). Although FTO has broad prospects as a therapeutic target for glioma, its specific efficacy still needs to be validated in further clinical trials. Another eraser, ALKBH5, is also highly expressed in gliomas. High expression of ALKBH5 significantly inhibits TME immune activation and promotes glioma growth. IOX1 is a specific inhibitor of ALKBH5. Pharmacological inhibition of ALKBH5 enhances the therapeutic efficacy of anti-PD-1 treatment in preclinical mouse models. Therefore, the combination of anti-PD-1 and anti-ALKBH5 is a promising therapeutic strategy for gliomas (96). Additionally, miR-1252-5p also serves as an inhibitor of ALKBH5, suppressing ALKBH5-mediated demethylation and thereby reducing NANOG expression in gliomas. Consequently, glioma tissues are more sensitive to TMZ, resulting in improved therapeutic outcomes (136).

There are many m6A readers that can be used as therapeutic targets. For example, miR-526b-3p can suppress IGF2BP1, thereby inhibiting the MAPK pathway and reducing the incidence of gliomas (88). Similarly, miR-4500 also targets IGF2BP1 to inhibit the development of gliomas (166). In addition to IGF2BP1, inhibition of IGF2BP2 increases the sensitivity of glioma cells to TMZ (113). Another study revealed that the lncRNA WEE2-AS1 can be methylated by m6A and recognized by IGF2BP3, which activates the PI3K-AKT signalling pathway, leading to the occurrence of glioma. They reported that blocking WEE2-AS1 expression improved the therapeutic sensitivity to dasatinib (72). Moreover, the inhibition of hnRNPC can reduce the expression of miRNA-21 in gliomas and promote the expression of PDCD4, thus inhibiting metastasis (120). Similarly, hnRNPA2/B1 inhibition is also expected to be a therapeutic target for gliomas (127). The IGF1/IGF1R inhibitor linsitinib preferentially targets YTHDF2-expressing cells, inhibiting GSC viability without affecting normal neural stem cells (NSCs) or impairing glioblastoma growth *in vivo (* 82).

In addition to directly inhibiting m6A readers, artificially reducing the upstream inducing factors of m6A readers can also be used for the treatment of glioma. High expression levels of some circRNAs, such as circNEIL3 and circHIPK3, promote the occurrence of glioma by enhancing the high expression of IGF2BP3 (90, 91). Artificially decreasing the expression of circRNAs is a potential prognostic biomarker and therapeutic target in glioma.

Currently, the application of RNA methylation in glioma treatment focuses primarily on alterations in m6A methylation levels. Most of these drugs are still in the preclinical development stage. Therefore, further exploration of glioma treatment through m6A methylation remains a primary research focus in the future. Another promising direction involves expanding trials using other forms of methylation for glioma treatment.

### 5.2 Targeting m5C

Although targeting m6A methylation remains the predominant approach for RNA methylation in glioma therapy, emerging research has identified RNA m5C methylation as a pivotal therapeutic target for the treatment of gliomas. miR-129-5p can inhibit DNMT3A, leading to significant cell cycle arrest, thereby suppressing the proliferation of glioma cells (167). The RNA decoys YBX1–1 and YBX1–2 specifically target YB-1, which prevents YB-1 from binding to the relevant mRNA and thus inhibits the activation of downstream oncogenes, resulting in slower tumour growth and better survival (130). Interestingly, highly expressed YB-1 can increase the oncolytic activity of XVir-N-31, which is a YB-1-dependent oncolytic adenovirus that has glioma-dissolving activity. Additionally, irradiation therapy before XVir-N-31 infection increases the migration of YB-1 into the nucleus, which significantly increases the oncolytic activity of XVir-N-31 (168). The combination of multiple treatment modalities and methylation therapy may become a hot research topic in the future. The role of TET in glioma is focused mainly on the demethylation of DNA by TET (169). TET converts the 5mC modification on DNA to 5hmC, which can inhibit the development of gliomas. However, high expression of SOX2 can inhibit the function of TET, thus promoting the development of glioma (170). This finding is consistent with the finding that highly expressed METTL3 in glioma increases SOX2 expression, thus inhibiting differentiation and promoting the generation of glioma (13). Therefore, efforts to decrease the expression of SOX2 and increase the expression levels of TET could be used to prevent the occurrence and progression of gliomas. Recent research has revealed that NSUN5 can recruit TET2, leading to conversion of the m5C modification to 5hmC on the CTNNB1 caRNA, which is then recognized by the reader RBFOX2, promoting caRNA degradation and immune system activation, increasing the phagocytosis of tumour-associated macrophages (TAMs) and facilitating the elimination of gliomas. Therefore, promoting NSUN5 expression has emerged as a novel therapeutic target for gliomas (171). Additionally, NSUN5 can facilitate the degradation of β-catenin mRNA, thereby enhancing the phagocytosis of TAMs (171). However, caution should be exercised in blindly increasing the expression levels of NSUN5, as studies have also reported its potential to promote the synthesis of a series of oncoproteins (156). The underlying reasons for this contradictory phenomenon await further investigation.

### 5.3 Targeting m1A

The application of m1A methylation therapy for treating gliomas is relatively rare. This suggests that the role of m1A in gliomas warrants further investigation. Nonetheless, we observed that TRMT6/61 promotes malignant transformation and progression by sustaining tRNA methylation in glioma. This process is inhibited by the protein kinase C PKCα (111). These findings suggest that PKCα might be used as a new target for the treatment of glioma.

In summary, the investigation of targeted RNA methylation therapy for glioma remains largely at the preclinical stage. While certain studies have exhibited the cytotoxic and inhibitory effects of these therapeutic agents on glioma cells *in vitro*, without inducing toxicity in normal cells, they usually lack comprehensive pharmacokinetic analyses (82, 115). Nonetheless, the potential of targeted RNA methylation therapy is substantial, particularly in light of the extensive clinical application of TMZ chemotherapy for gliomas characterized by high MGMT methylation, which theoretically enhances its feasibility. Consequently, future research exploring the combination of targeted RNA methylation therapy with TMZ chemotherapy represents a promising avenue for clinical investigation.

## 6 Conclusions and perspectives

In this review, we summarized the pathogenesis of glioma with respect to the RNA modifications of m6A, m5C, m7G, and m1A. Currently, there are more relevant studies on the role of RNA m6A modifications in the occurrence and development of glioma, whereas relatively few studies have investigated the roles of RNA m5C, m7G, and m1A modifications in the occurrence and development of glioma. We have summarized the changes in RNA methylation levels during glioma development and the associated downstream carcinogenic pathways. We note that most RNA methylations are elevated when glioma occurs. Reversing this alteration and maintaining cellular methylation homeostasis present vast therapeutic prospects. Additionally, detection of this alteration is helpful for predicting patient prognosis and detecting glioma progression.

In recent years, an increasing number of studies have focused on the mechanism of RNA methylation in the development of glioma. Excitingly, several advancements have been made. However, the effect of RNA methylation on glioma remains unclear, particularly the relationship between methylation content and the malignant grade of glioma. Some studies have found that high levels of methylation, such as METTL3-mediated m6A RNA methylation, can increase the incidence of glioma (13). However, some studies have found that high methylation levels can inhibit the progression of glioma, for example, inhibiting the expression of FTO after IDH mutation, resulting in increased methylation that degrades cancer-related mRNAs (165). Some studies have found that increased expression of hnRNPC is related to signalling pathways associated with the malignant degeneration of glioma (92, 120, 121). However, in another Lasso-Cox regression algorithm study, significantly increased hnRNPC expression was associated with a longer OS (150). Some studies have found that METTL3 stabilizes BUD13 and CDK12 mRNAs, subsequently leading to the phosphorylation of MBNL1, and thereby promoting the formation of VM in glioma (122). However, other studies have found that downregulation of METTL3 expression and upregulation of the expression of the eraser ALKBH5 can also promote VM (123). These opposing phenomena require further investigation.

We conclude that these contrasting results are partly due to the role of methylation readers. Some readers break down the methylated RNA, whereas others stabilize it. Due to the selection of various glioma models and the differing grades of glioma in the study, the baseline levels of RNA methylation in cells vary, resulting in divergent prognostic assessments regarding the detection of RNA methylation. Furthermore, the incorporation of studies utilizing diverse research and sequencing platforms introduces inherent systematic biases. To thoroughly assess the mechanisms of RNA methylation in glioma progression and its potential applications in diagnosis and treatment, a future systematic review or meta-analysis will be indispensable. According to the latest guidelines for gliomas, the classification of gliomas at the molecular level is becoming an increasingly accepted standard (3). Therefore, the study of RNA methylation should be synchronized with the molecular type of glioma. Specifically, the effects of RNA methylation alterations on the occurrence and development of glioma should be discussed according to its molecular type.

Our emphasis on the role of RNA methylation in gliomas is primarily informed by three key considerations: 1. Glioma stem cells (GSCs) play a pivotal role in therapeutic resistance and tumour recurrence. Recent research indicates that m6A RNA methylation exerts a dynamic regulatory effect on the equilibrium between self-renewal and differentiation in GSCs (76, 77, 79, 81–83) (**Figure 3**). This regulatory mechanism seems to be more pronounced in gliomas compared to solid tumours that do not possess a clearly defined stem cell hierarchy. 2. In the distinctively hypoxic microenvironment of gliomas, there is an upregulation of m6A methyltransferases, such as METTL3, which facilitates the adaptive translation of pro-survival mRNAs, including targets of HIF-1α (39, 87). This dependency on hypoxia-induced methylation is comparatively not obvious in cancers characterized by more vascularized microenvironments. 3. The existence of the blood-brain barrier presents a substantial obstacle to the utilization of diagnostic biomarkers and targeted therapies for gliomas. Currently, TMZ chemotherapy constitutes the primary clinical treatment for gliomas exhibiting high levels of MGMT methylation. Nevertheless, treatment alternatives are notably constrained for patients exhibiting resistance to TMZ. Considering the documented benefits of targeting RNA methylation to surmount drug resistance in gliomas (80, 109, 134–137)(**Table 1**), we posit that exploring RNA methylation in gliomas offers considerable potential for clinical application. Notably, the methylation level of RNA is significantly altered not only in glioma but also in other diseases, so this change may be used as a differential diagnostic marker (40). RNA methylation also plays an important role in promoting haematological tumours such as lymphoma and leukaemia and can be used as a potential diagnostic and therapeutic target. Therefore, we speculate that RNA methylation can be used as a novel biomarker in the differential diagnosis of central nervous system glioma and lymphoma.

In recent years, an increasing number of drugs have been developed based on RNA methylation (72, 113, 115, 168), but most are in the preclinical stage. However, glioma therapeutics based on RNA methylation clearly have broad prospects. In addition, recent research has found that miRNA-124-2, miRNA-135a-2, and miRNA-29a are the most effective miRNAs across all GBM subtypes with clinical relevance. High expression of all three miRNAs in GSCs significantly decreases GSC proliferation *in vitro (* 172, 173). Given that 2012 and 2015 studies reported that m6A methylation is involved in the modification of primary miRNAs, which results in alterations from primary miRNAs to miRNAs (69, 120), we believe that studying the modification of noncoding RNAs such as miRNAs might become a prospective research direction in the future.

## Supplementary material

Supplementary Table 1 Function implications of RNA methylation regulators.
