# Supplementary material for: The role of RNA methylation in glioma progression: mechanisms, diagnostic implications, and therapeutic value
Source: Front Immunol. 2025 May 21;16:1583039. doi: 10.3389/fimmu.2025.1583039 (PMC12133848; doi:10.3389/fimmu.2025.1583039)
Supplement: Supplementary Table 1 — Function implications of RNA methylation regulators. [file Table1.docx]

Supplementary table 1. Function implications of RNA methylation regulators

| **Methylation types** | **Regulators** | **Full term** | **Functional implications** |
| --- | --- | --- | --- |
| N6-methyladenosine (m6A) | METTL3 | Methyltransferase-like 3 | mRNA transcription start site and the 3′-untranslated region (UTR) m6A methylation |
|  | METTL14 | Methyltransferase-like 14 | mRNA transcription start site and the 3′-untranslated region (UTR) m6A methylation |
|  | WTAP | Wilms’ tumour 1-associating protein | The core structure of regulatory subunit m6A-METTL-associated complex |
|  | RBM15/RBM15B | RNA-binding motif protein15/15B | The core structure of regulatory subunit m6A-METTL-associated complex |
|  | VIRMA | VIR-like m6A methyltransferase associated | The core structure of regulatory subunit m6A-METTL-associated complex |
|  | ZC3H13 | Zinc finger CCCH-type containing 13 | Facilitating the nuclear localization of WTAP; Stretching the conformation of VIRMA |
|  | FTO | Fat mass and obesity-associated protein | N6,2’-O-dimethyladenosine (m6A_m_) demethylation |
|  | ALKBH5 | α-ketoglutarate-dependent dioxygenase alkB homologue 5 | N6-methyladenosine (m6A) demethylation |
|  | YTHDF | YTH domain family | RNA decay; Translation initiation |
|  | YTHDC | YTH domain-containing family | RNA splicing and nuclear transport |
|  | IGF2BP | Insulin-like growth factor 2 mRNA-binding protein | RNA stabilization; Translation initiation |
|  | EIF3 | Eukaryotic initiation factor 3 | Translation enhancement |
|  | hnRNP | Heterogeneous nuclear ribonucleoprotein | RNA splicing and nuclear transport |
|  | FMRP | Fragile X mental retardation protein | ﻿mRNA nuclear export |
| N5-methylcytosine (m5C) | DNMT2 | DNA methyltransferase 2 | DNA cytosine ring m5C methylation |
|  | NSUN | NOL1/NOP2/SUN domain | mRNA m5C modification (NSUN2) |
|  | TET | Ten-eleven translocation | N5-methylcytosine (m5C) oxidization and excision; 5-hydroxymethylcytosine (5hmC) production |
|  | ALYREF | ALY/REF export factor | mRNA nucleus export |
|  | YB-1 | Y box binding proteins 1 | RNA stabilization |
| N7-methylguanosine (m7G) | RNMT | RNA guanine-7 methyltransferase | mRNA m7G methylation; Cap homeostasis of the mRNA transcriptome |
|  | RAM | RNMT-activated small protein | Cofactor of RNMT |
|  | METTL1 | Methyltransferase-like 1 | tRNA m7G methylation; RNA stabilization |
|  | WDR4 | WD repeat-containing protein 4 | tRNA m7G methylation; RNA stabilization |
|  | WBSCR22 | Williams-Beuren syndrome chromosome region 22 | tRNA m7G methylation; RNA stabilization |
|  | TRMT112 | tRNA methyltransferase activator subunit 112 | tRNA m7G methylation; RNA stabilization |
| N1-methyladenosine (m1A) | TRMT10C | ﻿tRNA methyltransferase 10C | Mitochondrial ND5 mRNA methylation |
|  | TRMT6/61A | ﻿tRNA methyltransferase 6/61A | tRNA T-loop-like structures methylation |
|  | TRMT61B | ﻿tRNA methyltransferase 61B | Mitochondrial tRNA methylation |
|  | NML | Nucleomethylin | Mitochondrial ND5 mRNA methylation |
|  | ALKBH1 | α-ketoglutarate-dependent dioxygenase alkB homologue 1 | tRNA m1A demethylation |
|  | ALKBH3 | α-ketoglutarate-dependent dioxygenase alkB homologue 3 | mRNA m1A demethylation |
